# Metastatic Prostate Cancer Presenting as a Rectal Polyp: A Rare Occurrence

**DOI:** 10.7759/cureus.15115

**Published:** 2021-05-19

**Authors:** Ese Uwagbale, Ifeanyichukwu Onukogu, Vimal Bodiwala, Solomon Agbroko, Niket Sonpal

**Affiliations:** 1 Internal Medicine, Brookdale University Hospital Medical Center, Brooklyn, USA; 2 Gastroenterology and Hepatology, State University of New York Downstate Medical Center, Brooklyn, USA; 3 Obstetrics and Gynecology, Aspirus Keweenaw Hospital, Laurium, USA; 4 Obstetrics and Gynecology, Maimonides Medical Center, Brooklyn, USA; 5 Gastroenterology and Hepatology, Brookdale University Hospital Medical Center, Brooklyn, USA

**Keywords:** prostate cancer, malignant colonic polyp, rectal metastases, metastatic prostate carcinoma, rectal polyp

## Abstract

The prostate is anatomically located anterior to the rectum. Due to this proximity, locally advanced tumors of the prostate can invade the rectal tissue; likewise, colorectal cancers can invade the prostate gland; This presents mainly as an invasive mass with an identifiable primary and is rarely an isolated lesion. Prostate cancer rarely affects the gastrointestinal tract. Few cases of prostate cancer metastatic to the gastrointestinal tract have been reported in patients with a known prostate cancer history. Initial diagnosis of prostate cancer diagnosed from a colonic polyp is rare. We report a case of metastatic prostate cancer first diagnosed from a rectal polyp. Our patient is a 76-year-old man who initially presented with fatigue and 20 pounds weight loss in five months. The patient never had a colonoscopy before the presentation. A colonoscopy was done, which showed multiple colonic polyps and a pathology report of metastatic prostate cancer from a 12 mm rectal polyp.

## Introduction

Prostate cancer is the second most common cause of cancer death in men in the United States and the second most common cancer in men worldwide. In 2020, an estimated 191,930 newly diagnosed prostate cancer cases represented 10.6% of all new cancer cases [1]. 5.5% of all cancer deaths in 2020 were attributed to prostate cancer [1]. Prostate cancer rarely involves the colon. In a retrospective study of 74,826 patients with metastatic prostate cancer, only approximately 2.7% of the patients had metastasis to the gastrointestinal tract outside the liver [2]. The US Preventive Services Task Force (USPSTF) recommended screening methodology for prostate cancer is via prostate-specific antigen (PSA) measurement based on a shared decision between the patient and the doctor, which is controversial and not routinely recommended for all patients [3,4]. Confirmation of the diagnosis is by transrectal ultrasound-guided prostate biopsies (TRUS) [5,6]. The recommended screening age is 55-69 using PSA [6]. Prostate cancers may metastasize to bone, lungs, liver, and brain [7]. The most common site of metastasis is to bones and lymph nodes [2,8]. A few cases of metastatic prostate cancer found in colonic polyps have been reported in patients with a known history of prostate cancer [9]. We report a case of a new diagnosis of prostate cancer made from colonic polyp during diagnostic colonoscopy.

## Case presentation

**Figure 1:**
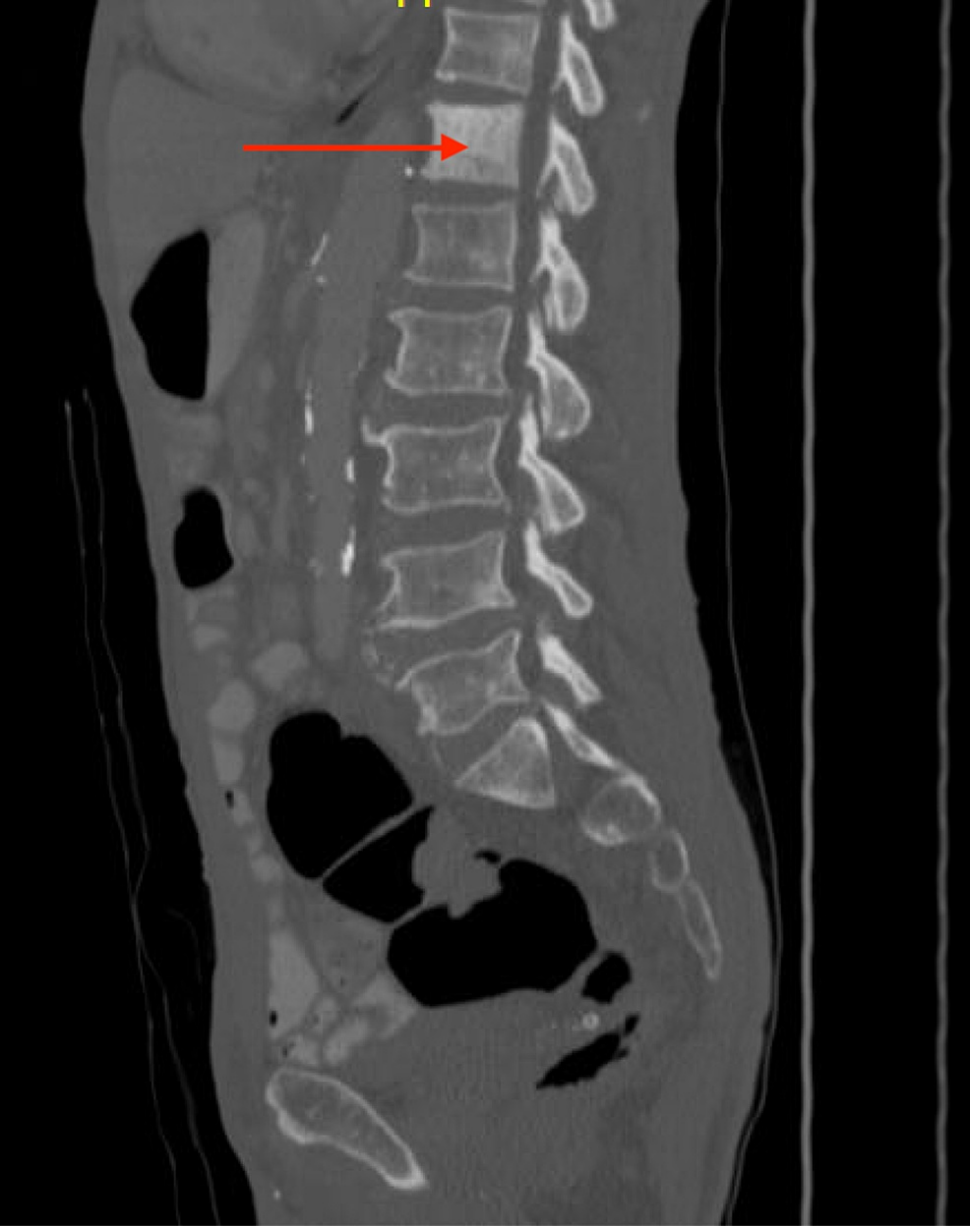
The arrow shows T12 sclerosis on CT scan.

**Figure 2:**
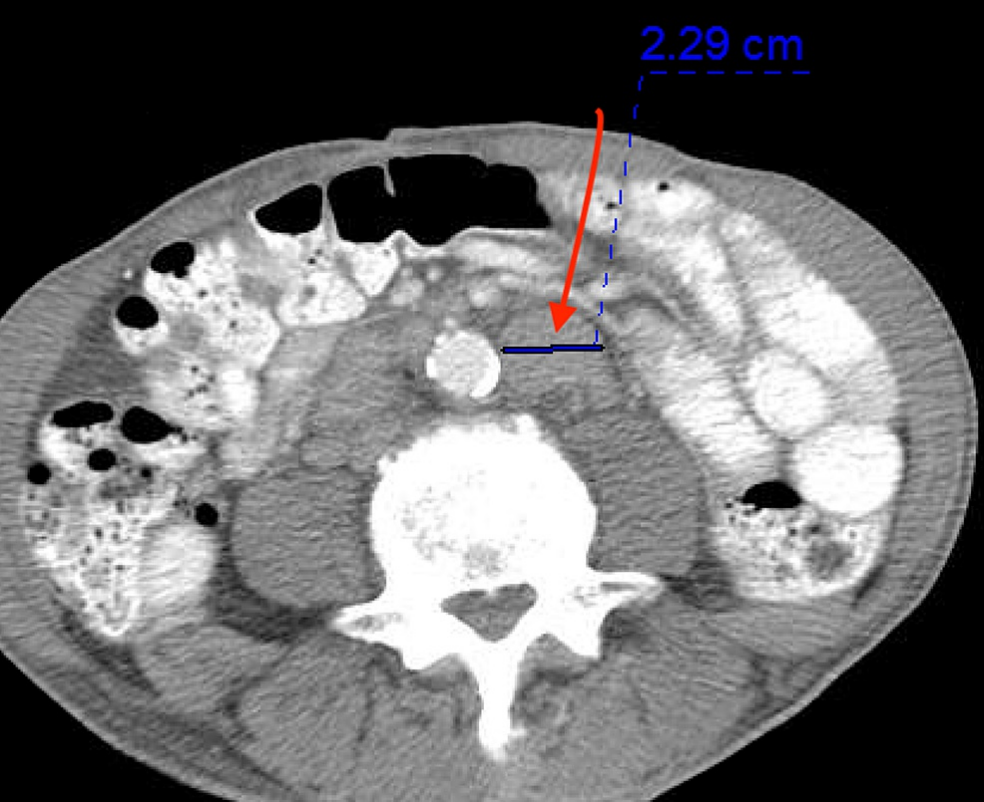
The arrow shows an enlarged lymph node on CT scan of the abdomen.

**Figure 3:**
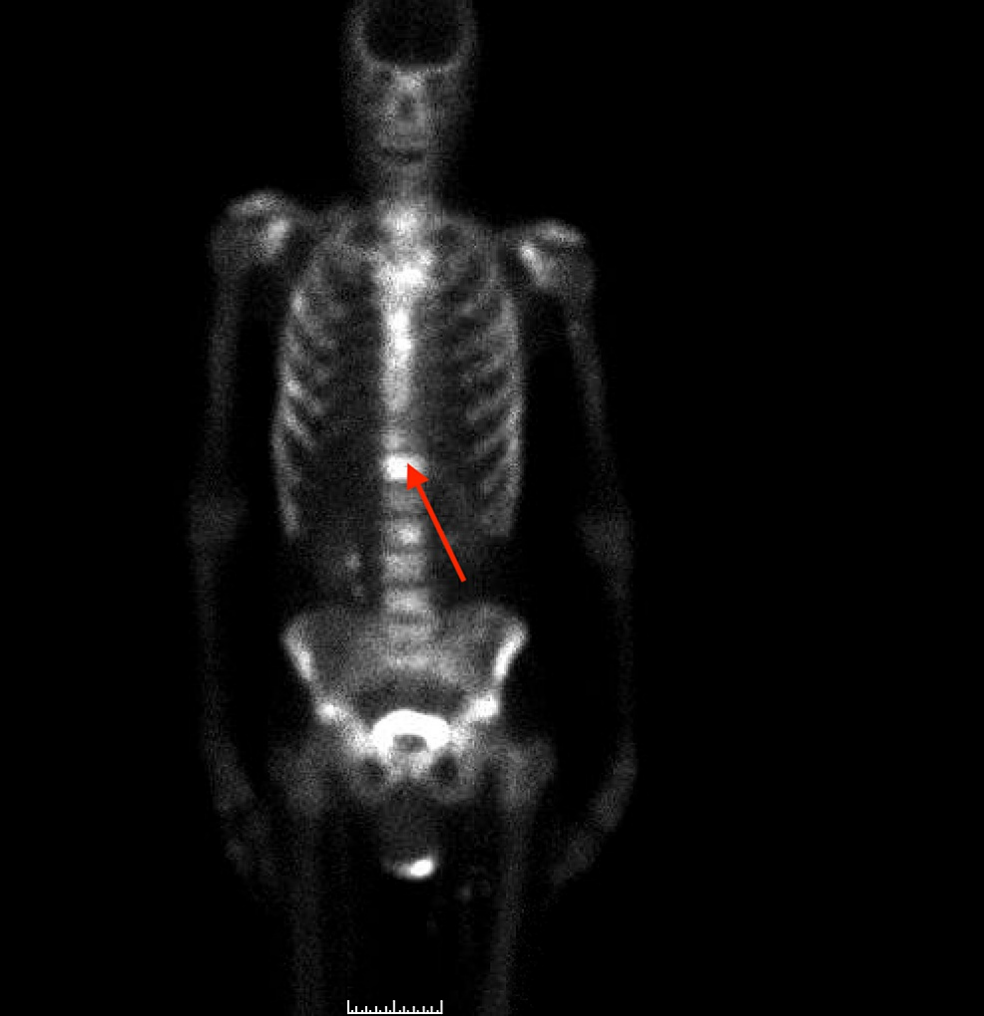
Bone scan showing multiple areas of increased uptake. The arrow points to increased uptake in T12.

A 76-year-old African American man, with no prior medical history, presented to the outpatient clinic with symptoms of unintentional weight loss of 20 Ibs in five months, fatigue, increased urinary frequency, back pain, and arthralgias. He had no family history of prostate or colon cancer. The patient had never had a colonoscopy. His labs were significant for elevated PSA of 568 ng/ml (reference range < 4ng/ml). CT scan of his chest and abdomen without contrast showed diffuse sclerotic lesions and indeterminate diffuse sclerosis of the T12 vertebral body (Figure 1). CT scan of his abdomen with oral and IV contrast showed enlarged upper abdominal, retroperitoneal, and pelvic lymph nodes, a T12 sclerotic lesion, and multiple tiny sclerotic lesions in the lower thoracic spine, lumbar spine, and pelvis suggestive of bony metastases (Figure 2). Colon's evaluation was limited on the CT due to constipation, and the prostate gland measured 4.5 cm transversely with no masses visualized within the prostate. Multiple foci of increased uptake were seen in T1 to T6, T12, left and right iliac bones, 7th, 8th, 9th right ribs, and the left 12th rib during a bone scan (Figure 3). 

**Figure 4:**
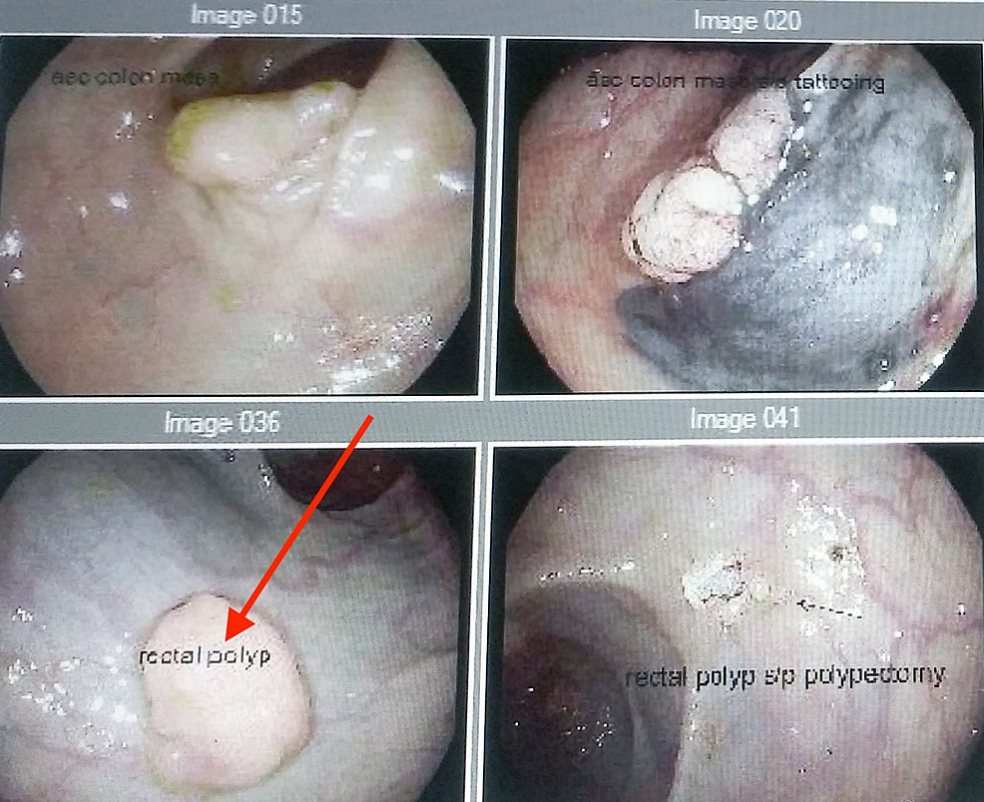
Colonoscopy showing multiple polyps. The arrow points to a 12 mm rectal polyp.

**Figure 5:**
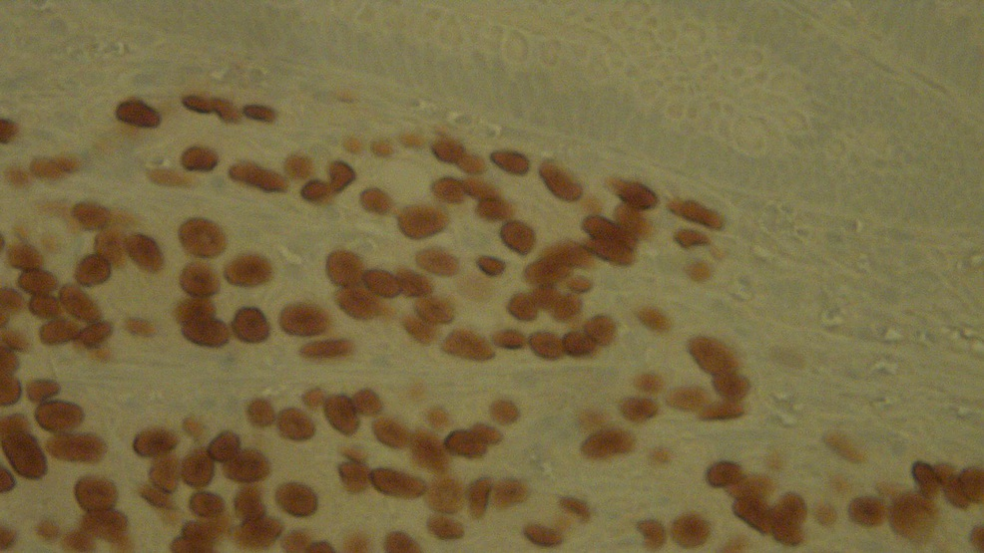
Immunohistochemical stain for NKX3.1 is positive in prostate adenocarcinoma and negative in adjacent colon tubular adenoma (40x). NKX3.1: homeobox protein Nkx-3.1

**Figure 6:**
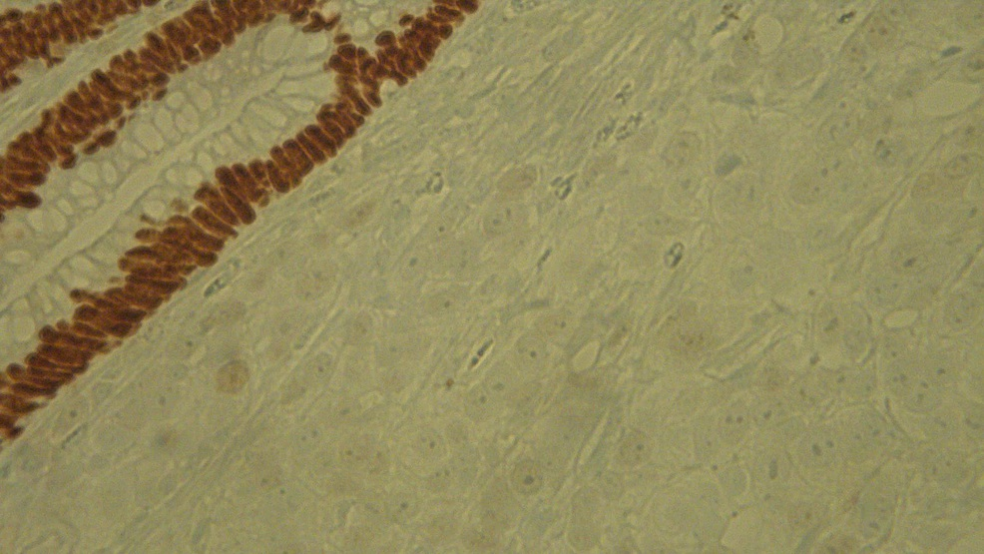
Immunohistochemical stain for CDX2 is positive in adenoma and negative in adjacent prostate adenocarcinoma (40x). CDX2: caudal type homeobox 2

Colonoscopy showed multiple polyps, including a 12 mm rectal polyp that was removed and retrieved (Figure 4). Immunohistochemical stains of rectal polyp for homeobox protein Nkx-3.1 (NKX3-1), α-methylacyl CoA racemase (P504S), and PSA were positive suggestive of prostate cancer as the primary origin (Figure 5). Caudal type homeobox 2 (CDX2) and monoclonal anti-carcinoembryonic antigen (mCEA), which are immunostains suggestive of colorectal origin, were negative (Figure 6). The findings confirmed colonic adenoma involved by prostatic adenocarcinoma with lymphovascular invasion. Biopsy of the retroperitoneal lymph node was positive for NKX3.1, prostatic-specific acid phosphatase (PSAP), and PSA, supporting the diagnosis of metastatic prostatic adenocarcinoma. The patient was treated with bicalutamide, leuprolide, and docetaxel.

## Discussion

The distinction between prostate cancer and colorectal cancer in a colonic biopsy can be a diagnostic challenge, especially in advanced stages. It is imperative to differentiate prostate cancer from colorectal cancer as the prognosis and treatment of these two cancers are very different. Prostate cancer preferentially metastasizes to the bone (80%) and lymph node (10.6%) [10]. Atypical metastasis sites include the liver, thorax, and, rarely, the digestive system [10]. The pathogenesis of this metastatic process is still not clearly understood, although "seed and soil," "homing," and "lymphatic spread" have all been suggested [10]. Histology and tumor markers help distinguish prostate cancer from other cancers. Histological identification of dirty necrosis and columnar cells with basal nuclei are seen mostly in colorectal cancer than prostate cancer [10]. Tumor markers such as PSA, NKX3.1, PSA, PSAP, P504S, and prostein (P501S) are primarily seen in prostate cancer, while B-catenin, cytokeratin 20 (CK20), and CDX2 are commonly seen in colorectal cancer [11,12]. In our case, the initial diagnosis of prostate cancer was made from a pathology report of a rectal polyp removed during a colonoscopy. The low frequency of prostate adenocarcinoma involving the rectum has been ascribed to the rectoprostatic fascia called the Denonvilliers' fascia, acting as a protective barrier against local spread [13]. Rectal involvement is possible through three different routes: direct invasion through Denonvilliers' fascia and rectal infiltration, lymphatic spread through the common pelvic lymph node channels, and implantation along a needle biopsy tract in rectal or perirectal tissue [14,15]. This patient did not have a history of a rectal needle biopsy or evidence of direct rectal invasion, suggesting metastasis via the lymphatic spread.

In a retrospective study in the literature by Tang et al., 9504 cases of rectal cancer were analyzed. Nine patients with prostate cancer and rectal wall invasion were misdiagnosed as rectal cancer [16]. The PSA level may help differentiate between prostate cancer and colorectal cancer, but it is nonspecific and not elevated in all prostate cancer patients. Elevated PSA values can occur in other tumors originating from the breast, salivary gland, and pancreas [10]. In high-grade prostatic cancers, PSA may lose sensitivity and appear negative [17]. For these reasons, we combine PSA with other tumor markers for increased sensitivity. In a clinical trial by Thompson et al., the prevalence of prostate cancer among 2950 men with PSA levels of 4 ng/ml or less was investigated; 15.2% of the patients were diagnosed with prostate cancer after a prostate biopsy [18]. In a case by Yoon et al., the diagnosis of metastatic prostate cancer was made from the pathology assessment of a rectal polyp in a patient with normal PSA and a significantly elevated carcinoembryonic antigen (CEA) level [19]. Rectal invasion of prostate cancer suggests a more advanced disease and portends a poor prognosis. Therefore, it makes it essential to make an accurate initial diagnosis to reduce mortality and morbidity. Colonoscopy should be considered in patients with prostate cancer without an identifiable metastatic site for a complete staging evaluation.

## Conclusions

Differentiating between advanced metastatic prostate and colorectal cancer from a colonic polyp can be a diagnostic challenge, especially in determining the primary site. Identifying the primary site is essential for proper staging and an appropriate treatment regimen. Therefore, it is vital to have a broad differential diagnosis when evaluating colonic polyps as there have been reported cases of extracolonic cancers first diagnosed from colonic polyps. The use of histological morphology and tumor markers has helped solve some of these difficulties. 

## References

[1] (2021). SEER: cancer stat facts: prostate cancer. https://seer.cancer.gov/statfacts/html/prost.html.

[2] Gandaglia G, Abdollah F, Schiffmann J (2014). Distribution of metastatic sites in patients with prostate cancer: a population-based analysis. Prostate.

[3] (2021). CDC: prostate cancer. https://www.cdc.gov/cancer/prostate/index.htm.

[4] Ilic D, Djulbegovic M, Jung JH (2018). Prostate cancer screening with prostate-specific antigen (PSA) test: a systematic review and meta-analysis. BMJ.

[5] Harvey CJ, Pilcher J, Richenberg J, Patel U, Frauscher F (2012). Applications of transrectal ultrasound in prostate cancer. Br J Radiol.

[6] Grossman DC, Curry SJ, Owens DK (2018). Screening for prostate cancer: US Preventive Services Task Force recommendation statement. JAMA.

[7] Zhao F, Wang J, Chen M (2019). Sites of synchronous distant metastases and prognosis in prostate cancer patients with bone metastases at initial diagnosis: a population-based study of 16,643 patients. Clin Transl Med.

[8] Wong SK, Mohamad NV, Giaze TR, Chin KY, Mohamed N, Ima-Nirwana S (2019). Prostate cancer and bone metastases: the underlying mechanisms. Int J Mol Sci.

[9] Nwankwo N, Mirrakhimov AE, Zdunek T, Bucher N (2013). Prostate adenocarcinoma with a rectal metastasis. BMJ Case Rep.

[10] Owens CL, Epstein JI, Netto GJ (2007). Distinguishing prostatic from colorectal adenocarcinoma on biopsy samples: the role of morphology and immunohistochemistry. Arch Pathol Lab Med.

[11] Gurel B, Ali TZ, Montgomery EA (2010). NKX3.1 as a marker of prostatic origin in metastatic tumors. Am J Surg Pathol.

[12] Jiang Z, Woda BA, Rock KL (2001). P504S: a new molecular marker for the detection of prostate carcinoma. Am J Surg Pathol.

[13] Sheridan T, Herawi M, Epstein JI, Illei PB (2007). The role of P501S and PSA in the diagnosis of metastatic adenocarcinoma of the prostate. Am J Surg Pathol.

[14] Galanopoulos M, Gkeros F, Liatsos C (2018). Secondary metastatic lesions to colon and rectum. Ann Gastroenterol.

[15] Vaghefi H, Magi-Galluzzi C, Klein EA (2005). Local recurrence of prostate cancer in rectal submucosa after transrectal needle biopsy and radical prostatectomy. Urology.

[16] Tang T, Yang Z, Zhang D, Qu J, Liu G, Zhang S (2017). Clinicopathological study of 9 cases of prostate cancer involving the rectal wall. Diagn Pathol.

[17] Zhu L, Luo C, Wu W, Ying J, Zhong H (2013). Prostate adenocarcinoma with negative immunohistochemical stain of prostate-specific antigen presenting with cervical mass: a case report. J Res Med Sci.

[18] Thompson IM, Pauler DK, Goodman PJ (2004). Prevalence of prostate cancer among men with a prostate-specific antigen level ≤4.0 ng per milliliter. N Engl J Med.

[19] Yoon G, Han MH, Seo AN (2019). Rectal invasion by prostatic adenocarcinoma that was initially diagnosed in a rectal polyp on colonoscopy. J Pathol Transl Med.

